# The N-Terminal Region of Middle East Respiratory Syndrome Coronavirus Accessory Protein 8b Is Essential for Enhanced Virulence of an Attenuated Murine Coronavirus

**DOI:** 10.1128/JVI.01842-21

**Published:** 2022-02-09

**Authors:** Yuming Li, Yingkang Jin, Lijun Kuang, Zhenhua Luo, Fang Li, Jing Sun, Airu Zhu, Zhen Zhuang, Yanqun Wang, Liyan Wen, Donglan Liu, Chunke Chen, Mian Gan, Jingxian Zhao, Jincun Zhao

**Affiliations:** a State Key Laboratory of Respiratory Disease, National Clinical Research Center for Respiratory Disease, Guangzhou Institute of Respiratory Health, the First Affiliated Hospital of Guangzhou Medical University, Guangzhou, China; b Pediatric Pulmonary Department, Guangzhou Women and Children's Medical Center, Guangzhou Medical University, Guangzhou, China; c Department of Clinical Laboratory, Guangdong Women and Children Hospital, Guangzhou, China; d Institute of Precision Medicine, The First Affiliated Hospital, Sun Yat-sen University, Guangzhou, China; e Institute of Infectious Disease, Guangzhou Eighth People's Hospital of Guangzhou Medical University, Guangzhou, China; Loyola University Chicago

**Keywords:** MERS-CoV, accessory protein 8b, virulence, innate immunity

## Abstract

Middle East respiratory syndrome coronavirus (MERS-CoV) is a beta coronavirus that emerged in 2012, causing severe pneumonia and renal failure. MERS-CoV encodes five accessory proteins. Some of them have been shown to interfere with host antiviral immune response. However, the roles of protein 8b in innate immunity and viral virulence was rarely studied. Here, we introduced individual MERS-CoV accessory protein genes into the genome of an attenuated murine coronavirus (Mouse hepatitis virus, MHV), respectively, and found accessory protein 8b could enhance viral replication *in vivo* and *in vitro* and increase the lethality of infected mice. RNA-seq analysis revealed that protein 8b could significantly inhibit type I interferon production (IFN-I) and innate immune response in mice infected with MHV expressing protein 8b. We also found that MERS-CoV protein 8b could initiate from multiple internal methionine sites and at least three protein variants were identified. Residues 1-23 of protein 8b was demonstrated to be responsible for increased virulence *in vivo*. In addition, the inhibitory effect on IFN-I of protein 8b might not contribute to its virulence enhancement as aa1-23 deletion did not affect IFN-I production *in vitro* and *in vivo*. Next, we also found that protein 8b was localized to the endoplasmic reticulum (ER)/Golgi membrane in infected cells, which was disrupted by C-terminal region aa 88-112 deletion. This study will provide new insight into the pathogenesis of MERS-CoV infection.

**IMPORTANCE** Multiple coronaviruses (CoV) cause severe respiratory infections and become global public health threats such as SARS-CoV, MERS-CoV, and SARS-CoV-2. Each coronavirus contains different numbers of accessory proteins which show high variability among different CoVs. Accessory proteins are demonstrated to play essential roles in pathogenesis of CoVs. MERS-CoV contains 5 accessory proteins (protein 3, 4a, 4b, 5, 8b), and deletion of all four accessory proteins (protein 3, 4a, 4b, 5), significantly affects MERS-CoV replication and pathogenesis. However, whether ORF8b also regulates MERS-CoV infection is unknown. Here, we constructed mouse hepatitis virus (MHV) recombinant virus expressing MERS-CoV protein 8b and demonstrated protein 8b could significantly enhance the virulence of MHV, which is mediated by N-terminal domain of protein 8b. This study will shed light on the understanding of pathogenesis of MERS-CoV infection.

## INTRODUCTION

Coronaviruses are positive-stranded RNA viruses that have the largest genomes of the known RNA viruses, typically 27–32 kb. Until now, seven human coronaviruses have been identified, including human coronavirus OC43 (HCoV-OC43), HCoV-NL63, HCoV-229E, HCoV-HKU1, severe acute respiratory syndrome coronavirus (SARS-CoV), Middle East respiratory syndrome coronavirus (MERS-CoV), and the novel coronavirus (SARS-CoV-2). The former four CoVs generally cause mild upper-respiratory-tract infection (1–4) and latter three could cause severe lower respiratory tract infections and even death (5). MERS-CoV was first identified in Saudi Arabia in 2012 (6), with the highest mortality rate among SARS-CoV, MERS-CoV, and SARS-CoV-2. By the end of June 2021, 2,574 confirmed cases and 886 deaths had been reported and the mortality rate was about 35% (7). Apart from nonstructure and structure proteins, each coronavirus encodes a set of accessory proteins that are interspersed between or within the structural proteins. The accessory proteins are various in numbers and functions and share lower sequence similarity in different coronaviruses. Generally, accessory proteins considered to be dispensable for viral replication; however, growing research showed these proteins play important roles in CoV pathogenesis (8–10).

MERS-CoV has five putative accessory proteins, including protein 3 (encoded by ORF3), protein 4a (encoded by ORF4a), protein 4b (encoded by ORF4b), protein 5 (encoded by ORF5), and protein 8b (encoded by ORF8b) (11). All four protein (protein 3, -4a, -4b, -5) deletions could attenuate MERS-CoV viral replication and augment host responses (8, 9). Plasmid transfection-mediated accessory protein overexpression could activate the inflammatory response or antagonize type I IFN response by various pathways. Several studies have shown that protein 4a, protein 4b and protein 8b can interfere with innate antiviral and proinflammatory responses *in vitro* (7, 10, 12–16). The protein 8b encoded by an internal ORF within the N gene via a ribosomal leaky scanning mechanism (11), which is similar to protein internal (I) in MHV, protein 9b in SARS-CoV, protein N2 in HKU1, and protein 9b in SARS-CoV-2 (17) SARS-CoV 9b can induce caspase-dependent apoptosis (18), or suppresses innate immunity by targeting mitochondria and the MAVS/TRAF3/TRAF6 signalosome (19). SARS-CoV-2 9b can inhibit IFN-I responses by targeting TOM70 (20, 21), or suppress IFN-I/III production induced by RIG-I/MDA5-MAVS signaling (22). MERS-CoV protein 8b can antagonize nuclear factor kappa B (NF-κB) activation or IFN-β promoter activation by luciferase reporter assay (23, 24). However, these results were generated from luciferase reporter assay and functional validation are required (25). Lok-Yin Roy Wong et al. found MERS-CoV protein 8b suppresses IFN-I expression by impeding the interaction of IKKε and HSP70 (7). However, since ORF8b is fully overlapping with nucleocapsid (N) protein gene, it would be difficult to generate ORF8b mutants in the context of MERS-CoV genome. Therefore, an alternative experimental approach is required to study the role of protein 8b in MERS-CoV infection. Mouse hepatitis virus (MHV) strain rJ2.2 is an attenuated neurotropic betacoronavirus that causes mild encephalitis (26). It contains 5 accessory genes, including NS2A, HE, ORF4, ORF5A an ORFI. Generally, accessory genes are unique in sequence and functions among different betacoronavirus (27). ORF4 in MHV was demonstrated to have no specific functions in MHV pathogenicity (28), which makes it a perfect alternative model for studying the roles of viral accessory proteins from the same genus, including SARS-CoV and MERS-CoV, by replacing this gene using a reverse genetic approach. No sequence homology between MERS-CoV accessory proteins and MHV viral proteins were found, suggesting it is unlikely that there will be functional redundancy between MHV and MERS-CoV accessory genes. Previously, Pewe and colleagues revealed that SARS-CoV protein 6 enhances viral virulence using the same MHV reverse genetic system (29).

Here, we constructed a set of recombinant MHV expressing individual MERS-CoV accessory proteins and demonstrated that accessory protein 8b could enhance viral replication *in vivo* and *in vitro*, as well as increase the mortality rate of infected mice. Protein 8b is 112 amino acids in length, with less than 30% similarity to any of structural or accessory proteins of MHV virus (by EMBOSS Needle tool). Protein 8b expression has also been detected in MERS-CoV infected cells using Western blot (WB). Further, we demonstrated that protein 8b could significantly inhibit type I interferon production (IFN-I) and innate immune response in mice infected with MHV expressing protein 8b. Protein 8b could initiate from multiple internal methionine sites and at least three protein variants were identified. Residues 1–23 of protein 8b was demonstrated to be responsible for increased virulence *in vivo*. We also found that protein 8b was localized to the endoplasmic reticulum (ER)/Golgi membrane in infected cells, which could be disrupted by C-terminal region deletion. This study will provide new insight into the pathogenesis of MERS-CoV infection.

## RESULTS

### Generation and screening of recombinant MHVs expressing MERS-CoV accessory proteins that had increased virulence.

**FIG 1:**
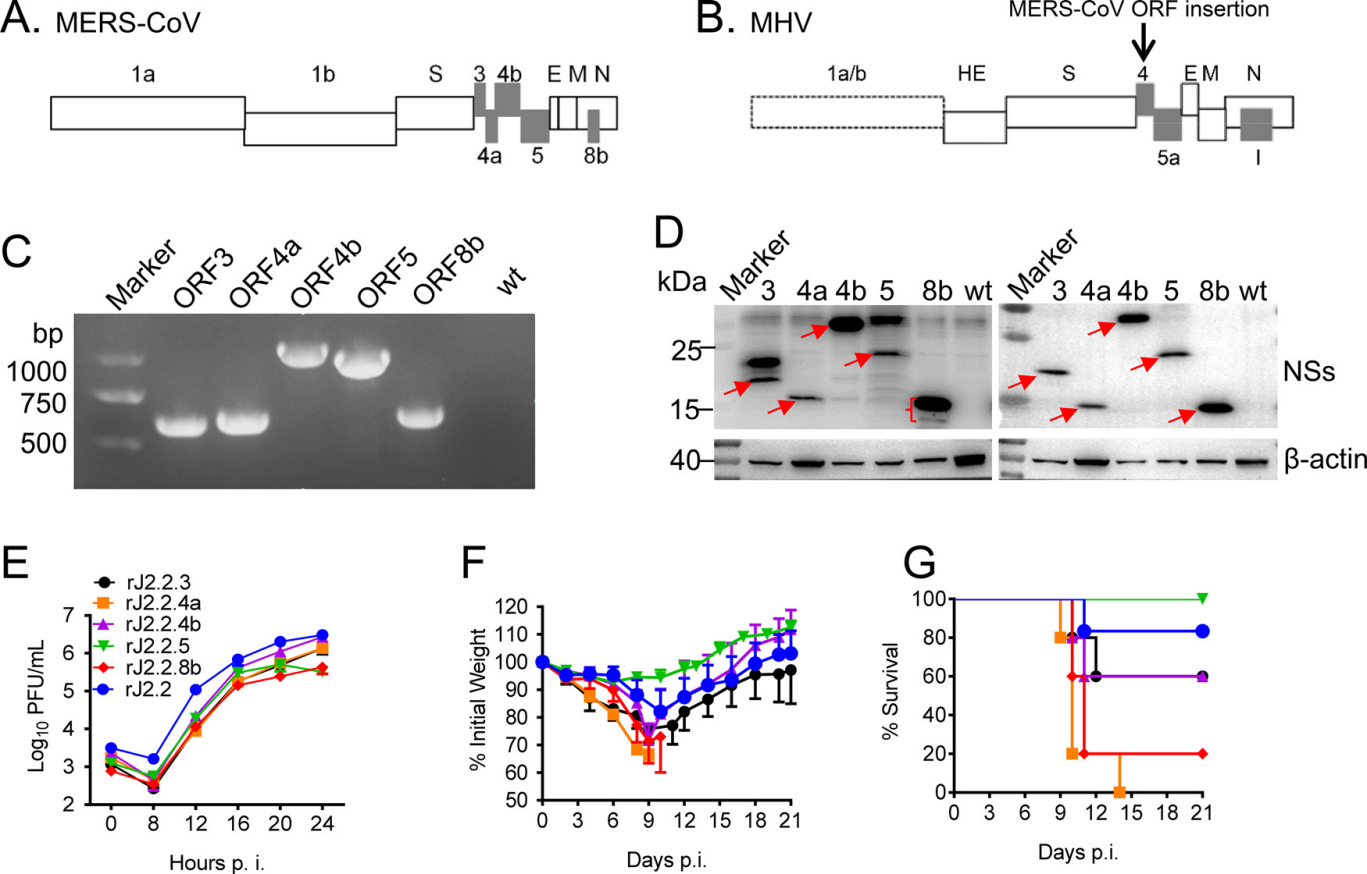
Generation and screening of recombinant MHVs expressing MERS-CoV accessory proteins that had increased virulence. (A) MERS-CoV genome with accessory proteins (gray). (B) MHV (rJ2.2) genome and MERS-CoV accessory was insert into rJ2.2 ORF4a sequence. (C) The inserted sequence of accessory proteins (ORF3a, -4a, -4b, -5, -8b) was detected by PCR. (D) 17CL-1 Cells were infected with wt-rJ2.2 or rJ2.2 expressing MERS-CoV accessory proteins at MOI 0.1 for 16 h. Accessory proteins were detected by WB analysis using an anti-HA antibody or an anti-Flag antibody. (E) 17CL-1 cells were infected with rJ2.2 and rJ2.2 expressing MERS-CoV accessory proteins at MOI 0.1. Supernatant was collected at indicated time points. Virus titers were measured in HeLa-MHVR cells. C57BL/6 mice were inoculated intracranially (i.c.) with 1,000 PFU of rJ2.2 or rJ2.2 expressing MERS-CoV accessory proteins. Weight loss (F) and survival rate (G) were monitored daily. Each group contained 5 mice. *, *P* < 0.05, the error bars indicate SEM.

**FIG 2:**
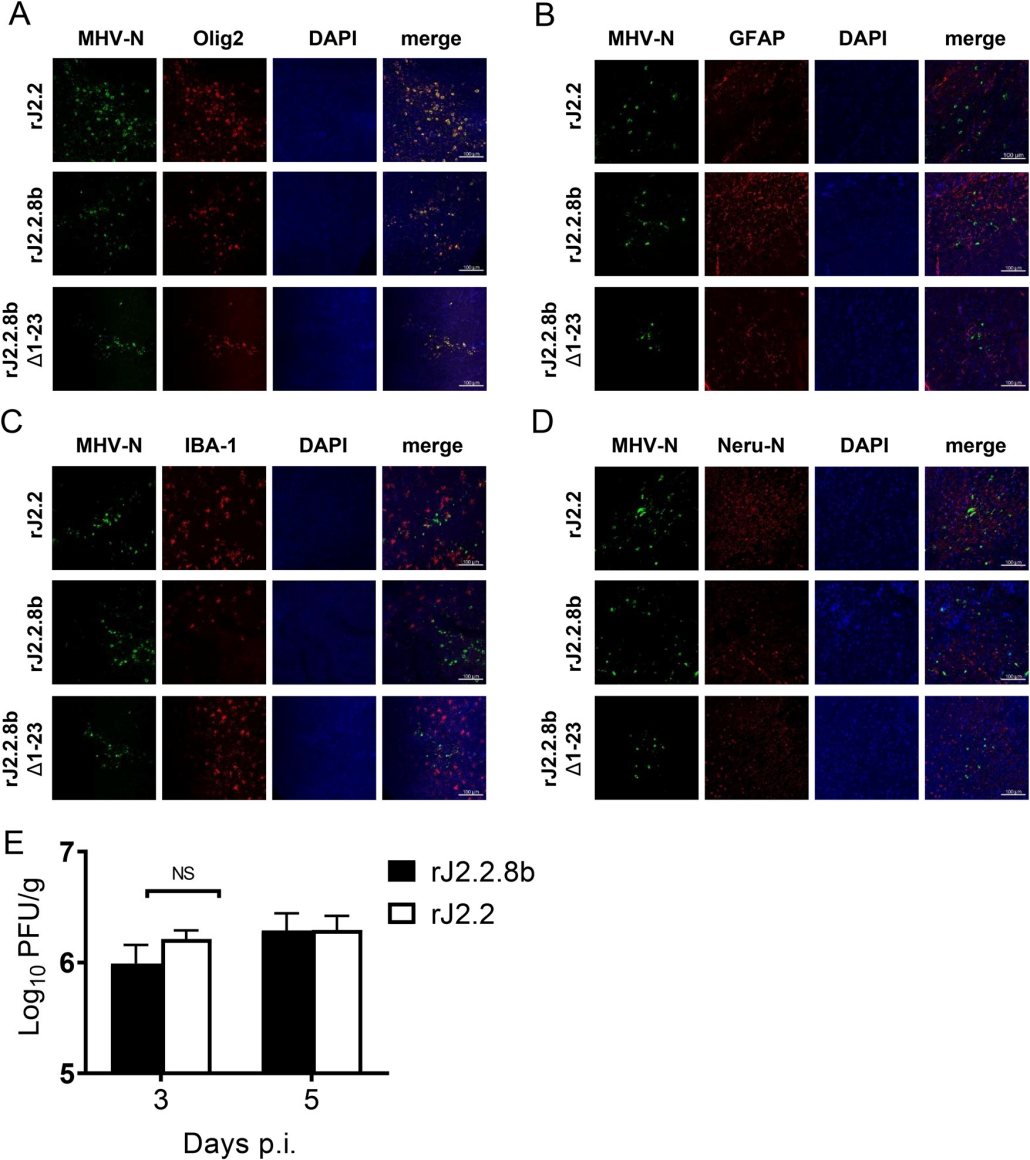
Cell tropism of rMHV infection in the brain. C57BL/6 mice were infected with 1,000 PFU of rJ2.2, rJ2.2.8b, and rJ2.2.8b Δ1-23. Mouse brains were harvested at 3 DPI, fixed in zinc formalin, and embedded in paraffin. Sections were stained with a mouse anti-MHV N protein antibody or a rabbit anti-HCoV-OC43-N antibody (green color) and (A) olig2 antibody (oligodendrocyte marker, red color), or (B) GFAP antibody (Astrocyte marker, red color), or (C) IBA1 antibody (microglia marker, red color), or (D) mouse anti-Neu-N antibody (neuron marker, red color). Nucleus was visualized by DAPI (blue color). Images were taken with a Zeiss model 880 laser-scanning confocal microscope (20×, bar = 100 μm). (E) C57BL/6 mice were infected with 1,000 PFU of rJ2.2 (*n* = 3) and rJ2.2.8b (*n* = 3) i.c. Mouse brains were collected at indicated time points. Virus titers were determined using HeLa-MHVR cells. The error bars indicate SEM.

MHV is a well-characterized neurotropic mouse coronavirus and offers several advantages for CoV studies as describe in introduction (29). So, we inserted each of the MERS-CoV accessory proteins gene (ORF3, ORF4a, ORF4b, ORF5, and ORF8b) into ORF4 of rJ2.2 (an attenuated neurotropic variant of MHV), respectively (Fig. 1A and B), as disruption of ORF4 of MHV does not affect its replication in cells nor cause acute encephalitis disease in mice (29). For subsequent detection, a Flag tag and an HA tag were inserted to the N- and C-end of each accessory protein, respectively. All insertions of the five proteins were confirmed by PCR and Western blot (WB) (Fig. 1C and D). Of note, multiple bands were observed in the lane of ORF8b in WB assay (Fig. 1D), which could result from multiple methionine sites in ORF8b sequence. We further characterized this phenomenon in Materials and Methods. To characterize recombinant virus replication, growth kinetics of these viruses were analyzed. As shown in Fig. 1E, all of the recombinant virus showed similar replication kinetics with slightly lower titers compared with wild type (wt) rJ2.2 virus. Next, C57BL/6 mice were challenged with 1000 PFU of each recombinant and WT rJ2.2 virus and clinical manifestations were monitored. As shown in Fig. 1F and G, rJ2.2.4a and rJ2.2.8b (rj2.2 expressing protein 4a or protein 8b) infections resulted in enhanced virulence in mice, but not recombinant viruses inserted with ORF3, ORF4b, and ORF5, as determined by more weight loss and greater mortality. Here, we chose rJ2.2.8b for further analysis since protein 4a has been extensively studied and several studies had reported that protein 4a significantly inhibits type I interferon (IFN-I) signaling by its dsRNA binding domain (14, 30). We also confirmed that rJ2.2.8b did not change cell tropism in the infected brain, as both rJ2.2, rJ2.2.8b mainly infected oligodendrocytes (Fig. 2). Of note, the virus titers showed no significant difference between rJ2.2 and rJ2.2.8b in infected mouse brains (Fig. 2E).

### Protein 8b enhanced virus replication and increased viral protein accumulation in vitro.

**FIG 3:**
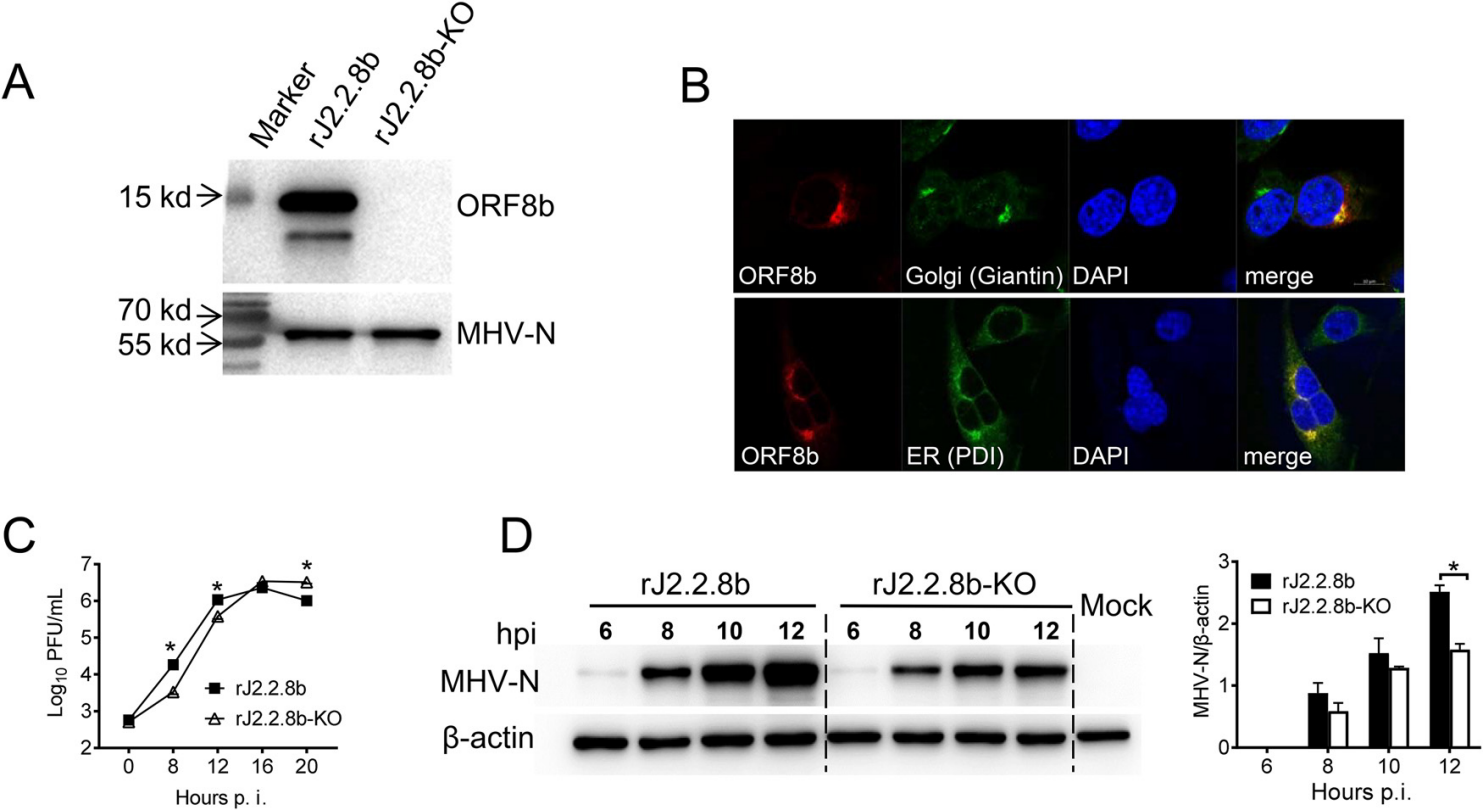
Protein 8b enhanced virus replication and increased viral protein accumulation *in vitro*. (A) As a control, MERS-CoV accessory protein ORF8b-KO sequence (the start codon ATG was mutated to AAA, another ATG at 154–156 was mutated to termination codon TAA) was introduced into rJ2.2 ORF4a as described in Materials and Methods. protein 8b expression in 17Cl-1 cells after recombinant virus infection was detected by Western blotting assay using an anti-HA tag antibody. (B) Subcellular localization of protein 8b in virus-infected cells. 17CL-1 cells were infected with rJ2.2.8b at 0.1 MOI and fixed at 12 h p.i. After Triton X-100 permeabilizations, cells were stained with mouse anti-HA-tag antibody and rabbit anti-Giantin (Golgi marker) antibody or rabbit anti-PDI (ER marker) antibody. Nucleus was visualized by DAPI. Images were taken with a Zeiss model 880 laser-scanning confocal microscope. (C) 17CL-1 cells were infected with the indicated virus for 1 h at MOI 0.1. Cells were then rinsed and replaced with fresh DMEM containing 2% FBS. Supernatant was collected at indicated time points and titrated on HeLa-MHVR cells. (D) 17Cl-1 cells infected with indicated virus were harvested and lysed. Viral N protein was detected by Western blotting using a mouse anti-MHV-N antibody.

In order to control the influence of exogenous sequence insertion into MHV genome, we developed another recombinant virus in which a mutated start codon of ORF8b together with an additional stop codon introduced at residue 25. This virus was designated rJ2.2.8b-KO. The KO mutation was confirmed by WB (Fig. 3A). Of note, multiple bands were frequently observed in the lane of rJ2.2.8b in WB assay when using anti-HA antibody (C-terminal) (Fig. 3A), but never found using Flag antibody (N-terminal) (Fig. 1D), which could result from multiple methionine sites in ORF8b sequence. We further characterized this phenomenon in Fig. 6. Previous studies had shown that MERS-CoV accessory proteins have different subcellular localization (10). Immunofluorescence analysis (IFA) revealed that the protein 8b was colocalized with endoplasmic reticulum (ER) and Golgi compartments in infected 17CL-1 cells (Fig. 3B).

To compare growth kinetics, 17CL-1 cells were infected with rJ2.2.8b and rJ2.2.8b-KO at MOI = 0.1. Supernatants were harvested at indicated time points postinfection (p.i.). As shown in Fig. 3C, J2.2.8b virus replicated to higher titer in infected cells than rJ2.2.8b-KO virus before reaching the plateau at 16 h. p.i., suggesting that protein 8b promoted virus replication at early stage of infection. This result was further confirmed by WB analysis of viral Neucleocapsid protein (N) accumulation in infected 17Cl-1 cells by WB (Fig. 3D). N protein accumulated to higher levels at 10–12 h after rJ2.2.8b infection, compared with rJ2.2.8b-KO (Fig. 3D), which was consistent with virus replication results in the cell supernatants (Fig. 3C).

### Protein 8b increased morbidity and mortality of rJ2.2.8b infected mice.

**FIG 4:**
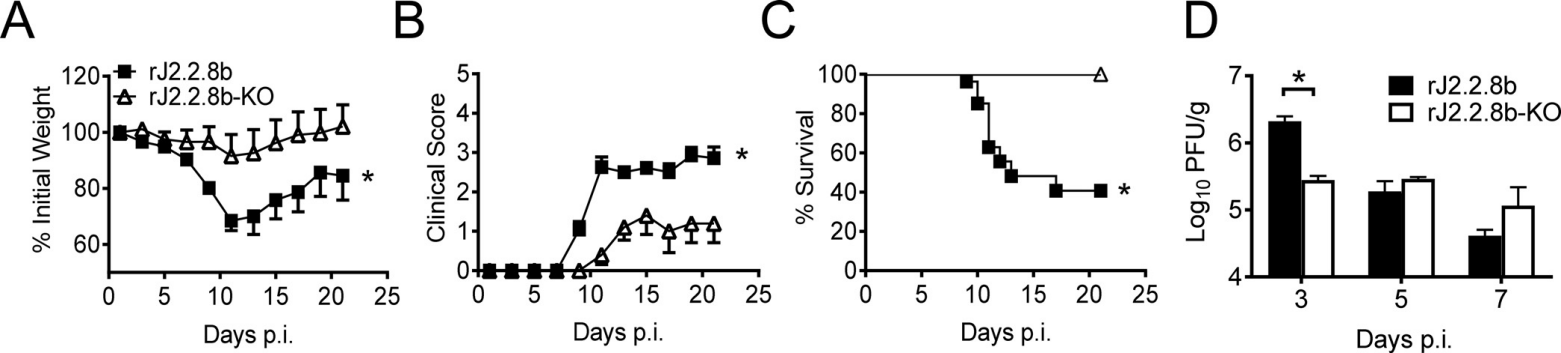
Protein 8b increased morbidity and mortality of rJ2.2.8b infected mice. C57BL/6 mice were infected with 1,000 PFU of rJ2.2.8b and rj2.2.8b-KO i.c. and monitored for weight loss (A), clinical sign (B), and mortality (C). The clinical sign scores were described in materials and methods. groups of 10 infected mice in each group were analyzed in two independent experiments. (D) Brains of mice infected with J2.2.8b (*n* = 4 or 5) or J2.2.8b -KO (*n* = 4 or 5) were collected at indicated time points. virus titers were determined using hela-mhvr cells. *, *p* < 0.05, the error bars indicate sem.

Consistent with Fig. 1F and G, mice infected with rJ2.2.8b lost more body weight and exhibited greater morbidity as determined by clinical scores than rJ2.2.8b-KO infected mice (Fig. 4A and B). More than 60% of mice died by day 17 post rJ2.2.8b infection. In contrast, all mice infected with rJ2.2.8b-KO survived until 21 days. p.i. (Fig. 4C). Consistent with these results, viral titers were significantly higher in the brains of rJ2.2.8b infected mice than those of rJ2.2.8b-KO at 3 days p.i. (Fig. 4D). These results demonstrated that protein 8b from MERS-CoV could significantly enhance the virulence in MHV infected mice.

### Protein 8b suppressed innate immune response after rJ2.2.8b infection *in vivo* and *in vitro*.

**FIG 5:**
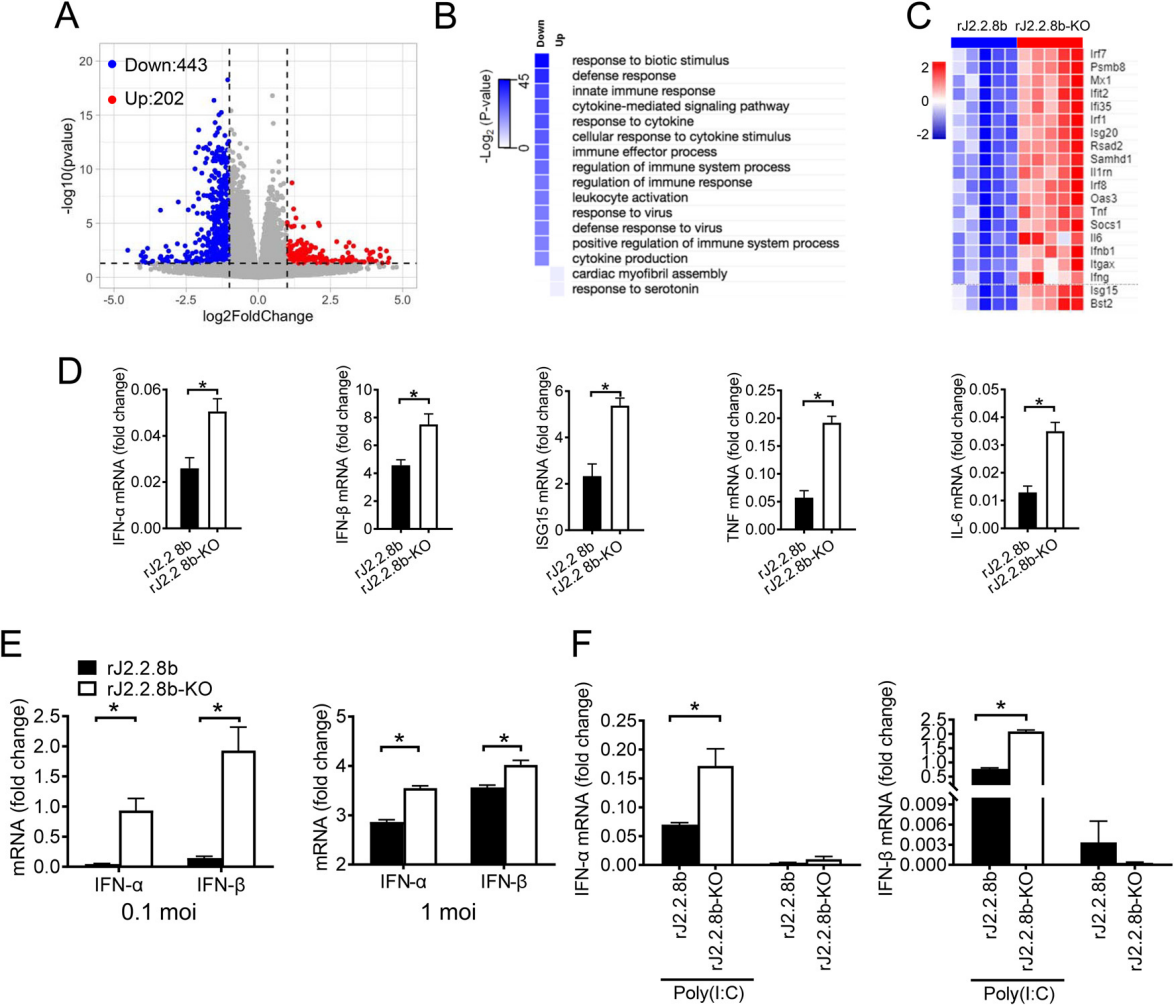
Protein 8b suppressed innate immune response after rJ2.2.8b infection *in vivo* and *in vitro*. C57BL/6 mice were infected with 1,000 PFU of rJ2.2.8b (*n* = 5) or rJ2.2.8b-KO (*n* = 5) i.c. Brains were harvested at 3 days p.i. Total RNA was extracted using TRIzol reagent, and RNA-seq analysis was performed using Illumina Hiseq machine. The differentially expressed genes were analyzed. (A) Volcano plot showing differentially expressed genes in the brains of mice infected with rJ2.2.8b compared to mice infected with rJ2.2.8b-KO. A total of 645 genes were differentially regulated. (B) Gene ontology (GO) analysis and (C) heat map of differentially expressed genes. (D) Five DEGs associated with innate immunity in RNA-seq were confirmed by real-time-PCR (RT-PCR). (E) Protein 8b inhibited IFN-I production in infected bone marrow-derived macrophages (BMM). BMM were infected with indicated virus at 0.1 or 1 MOI. Total cellular RNAs were extracted from individual cultures at 16 h p.i. using TRIzol reagent. IFN-I mRNA was quantified by real-time PCR as described in Materials and Methods. (F) Protein 8b inhibited IFN-I production induced by poly(I·C) treatment. L929 cells were infected with indicated virus for 12 h. Cells were then transfected with 2.5 μg/ml poly(I·C). Four hours later, cells were harvested into TRIzol. mRNA was extracted and analyzed by RT-PCR. *, *P* < 0.05, the error bars indicate SEM.

As significant differences in viral titers were observed at 3 days. p.i., but not at following time points (Fig. 4D), we proposed that the protein 8b might affect innate immune pathways at early stage of infection. RNA-seq was performed using brain samples harvested at 3 days p.i. Differentially expressed genes (DEGs) in the brain tissues of mice infected with rJ2.2.8b or rJ2.2.8b-KO were compared. We found 645 DEGs containing 202 upregulated and 443 downregulated genes in rJ2.2.8b groups compared with rJ2.2.8b-KO groups (Fig. 5A). Most downregulated genes were associated with innate immune pathways and inflammation pathways, including interferon stimulated genes (ISGs) and inflammatory cytokines in rJ2.2.8b infected mice (Fig. 5B and C). Several key genes were validated by real-time RT-PCR analysis. As shown in Fig. 5D, IFN-I, ISG15, TNF, and IL-6 were significantly downregulated in rJ2.2.8b infected mice, indicating protein 8b efficiently inhibited antiviral innate response. Further, rJ2.2.8b infected bone marrow-derived macrophages (BMM) also produced less IFN-I *in vitro* as determined by real-time RT-PCR, which were consistent with RNA-seq data *in vivo* (Fig. 5E). A previous study showed that fibroblasts cells did not produce IFN-I after MHV infection (31). We next examined whether poly(I·C) treatment could reverse this effect in rJ2.2.8b infected cells. The inhibitory effect of protein 8b on poly(I·C) induced IFN-I production was analyzed in L929 cells. Cells were infected with rJ2.2.8b or rJ2.2.8b-KO for 12 h, and then transfected with poly(I·C) for 4 h. Consistent with previous report, rJ2.2 infection alone did not induce significant IFN-I production (Fig. 5F). Although poly(I·C) treatment resulted in higher IFN-I production in both rJ2.2.8b or rJ2.2.8b-KO infected cells, IFN-I production in rJ2.2.8b infected cells still lower than rJ2.2.8b-KO infected cells (Fig. 5F). Together, these results suggested that protein 8b inhibited innate immune response after MHV infection.

### The N-terminal region of protein 8b was essential for enhanced viral virulence.

**FIG 6:**
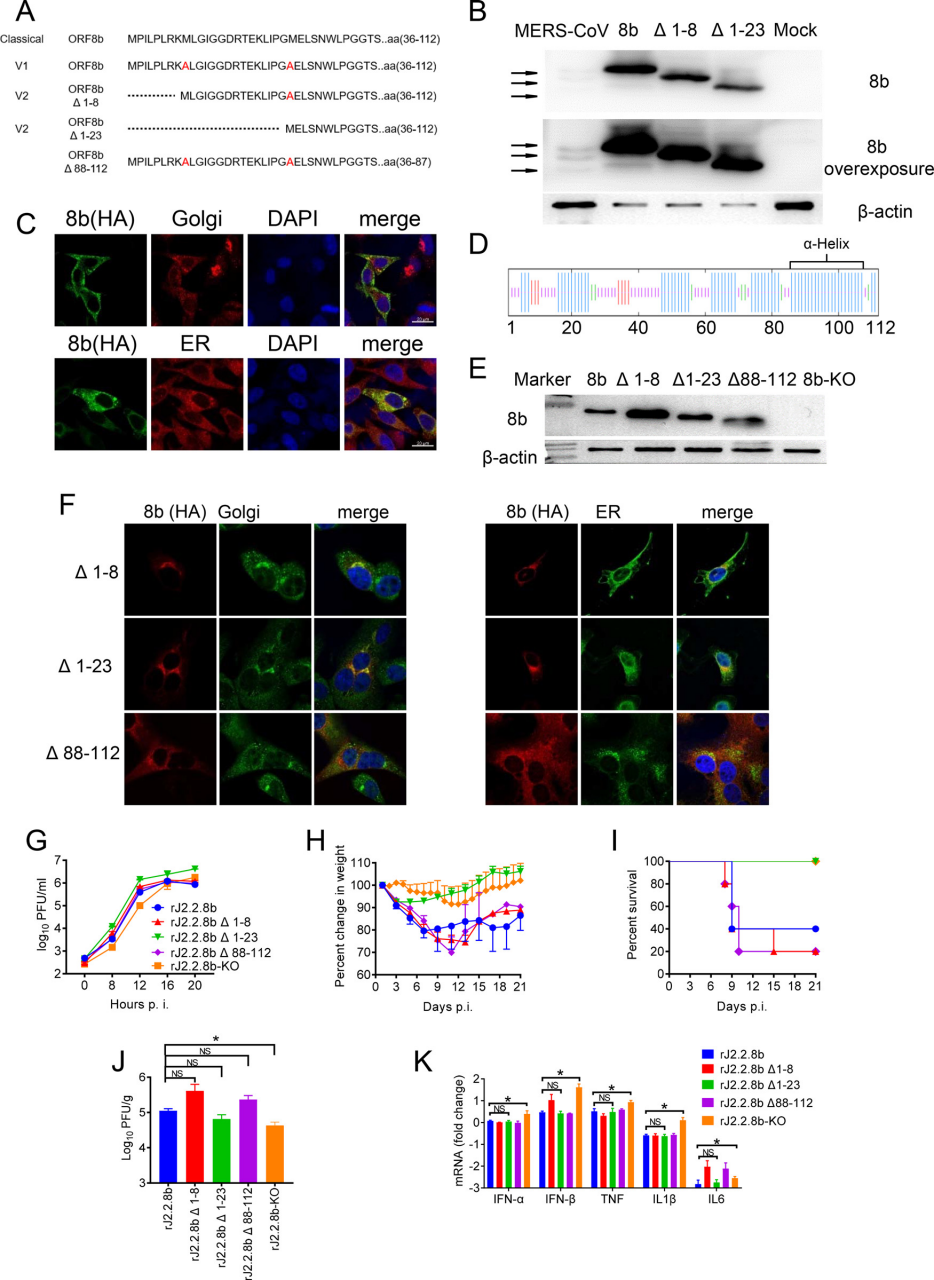
The N-terminal region of ORF8b was essential for enhanced viral virulence. (A) The sequences of protein 8b mutants or deletions with the C-terminal region. (B) protein 8b mutants were detected by Western blotting using an anti-protein 8b antibody (with an overexposed image), lane 1–5: lysate of Vero cells infected with MERS-CoV, lysates of 293T cells transfected with pCAGGS-ORF8b,-ORF8b Δ1-8, or –ORF8b Δ1-23, and lysate of uninfected Vero cells. (C) 17CL-1 cells were transfected with pCAGGS-ORF8b-HA using lipofectamine 2000 transfection reagent. After 36 h, cells were fixed and stained with mouse anti-HA-tag antibody and rabbit anti-Giantin (Golgi marker) antibody (1:200, Biolegend) or rabbit anti-PDI (ER marker) antibody (1:50, CST). Nucleus was visualized by DAPI. Images were taken with a Zeiss model 880 laser-scanning confocal microscope (100×, bar = 20 μm). (D) protein 8b sequence. Secondary structure of OFR8b was predicted using SOPMA (https://npsa-prabi.ibcp.fr/cgi-bin/npsa_automat.pl?page=npsa_sopma.html), Helix: blue; Coil: purple; Sheet: red; Beta turn: green. (E) rJ2.2.8b mutants were detected by Western blotting using an anti-HA antibody. (F) 17CL-1 cells were infected with rJ2.2.8b mutants at 0.1 MOI, and fixed at 12 h p.i. Cells were permeabilized with Triton X-100. protein 8b mutants were detected using mouse or rabbit anti HA-tag antibody and rabbit anti-Giantin antibody or rabbit anti-PDI antibody. Nucleus was visualized by DAPI. Images were taken with a Zeiss model 880 laser-scanning confocal microscope. (G) 17CL-1 cells were infected with virus at MOI 0.1. Supernatant was harvested at the indicated times, and virus titers were determined by plaque assay in HeLa-MHVR. C57BL/6 mice were infected with 1000 PFU of indicated virus i.c. Weight loss (H) and mortality (I) were monitored Virus titers (J) in the brains were determined on 3 days p.i. Each group included five mice. (K) C57BL/6 mice were infected with 1000 PFU of indicated virus i.c. IFN-IFN-α, IFN-β, TNF, IL1β and IL-6 mRNA transcription levels in the brains of mice infected with indicted virus were shown. *, *P* < 0.05, the error bars indicate SEM.

As shown in Fig. 1D and Fig. 2A, protein 8b potentially encodes multiple variants since it contains three methionines in its sequence (Fig. 6A). To determine which variant is responsible for enhanced virulence, several recombinant rJ2.2 (rJ2.2.8b Δ1-8 and rJ2.2.8b Δ1-23 with a Flag-tag at the N-end and an HA-tag at C-end of protein 8b), as well as eukaryotic expressing plasmids (without any tags which reflected the real sizes of protein 8b variants) inserted with truncated ORF8b were generated, where methionnines were replaced with alanines (Fig. 6A). Protein sizes of truncated protein 8b were analyzed by WB using rabbit polyclonal serum against protein 8b (Fig. 6B). First, multiple variants of protein 8b were confirmed using authentic MERS-CoV infected cell lysate. The protein size from cells transfected with full-length protein 8b was identical with the protein 8b band with the highest molecular weight in MERS-CoV infected cells. While protein 8b bands from 8b Δ1-8 and 8b Δ1-23 transfected cells were consistent with the two lower molecular weight bands, indicating protein 8b could initiate from internal methionines and encode three truncated protein 8b (Fig. 6B).

Our result showed that protein 8b colocalize with ER and Golgi in plasmid transfected 17Cl-1 cells (Fig. 6C). Further sequence analysis of protein 8b suggested that it contains an α- helix C-region which might contribute its subcellular localization (Fig. 6D). Next, we also generated another rJ2.2 with aa 88–112 deletion (rJ2.2.8b Δ88-112) (Fig. 6A). Truncated protein 8b expressions were confirmed by WB, and IFA assay using infected 17Cl-1 cells (Fig. 6E and F). As shown in Fig. 6F, the N-terminus deletion did not alter protein 8b subcellular localization, which was consistent with full-length protein 8b (Fig. 3B), but Δ88-112 deletion ablated its ER and Golgi localization, which was localized in cytoplasma of 17Cl-1 cells.

To further determine the role of these protein 8b variants and deletions on virulence,17Cl-1 cells were infected with truncated rJ2.2.8b viruses, respectively. Virus titers were determined and compared. All recombinant viruses exhibited similar growth kinetics. The titer of rJ2.2.8b Δ1-23 was slightly higher than the other viruses (Fig. 6G). To determine the role of protein 8b truncations *in vivo*, mice were infected with these mutant recombinant viruses and weight loss and survival rates were monitored. As shown in Fig. 6H and I, mice infected with rJ2.2.8b Δ1-8, rJ2.2.8b Δ88-112, and rJ2.2.8b displayed similar weight loss with 60–80% mortality in mice. However, the mice infected with rJ2.2.8b Δ1-23 were all survived (Fig. 6I). Viral titers in the brains of infected mice were measured at 3 days. p.i. As shown in Fig. 6J, no significant difference was observed between rJ2.2.8b and rJ2.2.8b Δ1-23, although rJ2.2.8b Δ1-23 infection showed slightly lower viral replication in the brain. These results indicated that the aa 1–23 region of protein 8b was essential for enhanced virulence in the attenuated murine coronavirus. To determine whether aa 1–23 deletion impairs the inhibitory function of protein 8b on IFN-I production, IFN-I mRNA levels in the brains of mice infected with rJ2.2.8b variants were analyzed. No significant difference of IFN-I mRNA levels was detected, suggesting that aa 1–23 domain was not responsible for the inhibition of IFN-I production of protein 8b (Fig. 6K).

## DISCUSSION

MERS-CoV could led to acute pneumonia with renal failure (6) and also has the highest fatality rate among all human respiratory coronaviruses according to WHO. MERS-CoV genome encodes a variety of accessory proteins which has no significance sequence homology even among closely related CoVs. The role of accessory proteins on the innate immune response has been investigated in some coronaviruses. Most of them play important roles in inhibition of IFN-I response or facilitate the inflammatory response. Previous studies showed that the MERS-CoV accessory protein 4a can suppress IFN-I production (13) and inhibit PKR-mediated antiviral response (14). Protein 4b inhibits NF-κB-dependent innate immune response (12), protein 8b inhibits NF-κB signaling (24) or type I IFN expression (7, 24). However, most of these studies were performed by eukaryotic plasmid overexpression. Some recent studies utilizing recombinant MERS-CoV have elucidated the functions of some of these proteins, such as protein 4a, protein 4b, and protein 5 (32–34). Although MERS-CoV protein 8b suppresses IFN-I expression in MERS-CoV-Δ8b (protein 8b knockout) infected cells (18), little is known about the role of protein 8b on viral virulence *in vivo*.

When absence of all four accessory proteins (protein 3, -4a, -4b, and -5), MERS-CoV attenuated in mice (8), indicating accessory proteins might play important roles on virulence enhancement. However, only deletion of protein 5 increase the virulence of a mouse-adapted MERS-CoV strain (33). In order to reduce the functional redundancy or compensatory between different proteins on the virulence of the virus, we constructed series of recombinant rJ.2.2 viruses inserted with only one MERS-CoV accessory protein. In order to ensure the transcription of ORFs, an MHV traditional TRS (transcription regulating sequence) sequence was added before the ORFs. Our previous study also found the insertion of exogenous genes could slightly reduce rJ2.2 replication, so we constructed the corresponding KO virus as control. Thornbrough J.M. et al., demonstrated that MERS-CoV protein 4b could increase MHV replication *in vitro* and *in vivo* by inhibiting host RNase L Activation (15). However, in this study, no weight change and survival rate of mice infected with MHV-ORF 4b recombinant virus were provided. Based on our results in Fig. 1, protein 4b increased rMHV virulence as determined by increased mortality rate after infection. Another studies described that MERS-CoV protein 4a increases recombinant encephalomyocarditis virus (EMCV) replication efficiency by suppressing the PKR-dependent stress response pathway (14), which is consistent with our results. All of the mice infected with rJ2.2.4a succumbed to the infection (Fig. 1). In this study, we demonstrated that two MERS-CoV accessory proteins, protein 8b could enhance viral virulence in MHV infected mice as determined by increased weight loss and mortality rate. *In vitro* growth curve showed that rJ.2.2.8b infection could produce more progeny virus at the early phase of infection and significant differences in titers in mouse brains were also observed in the early time of infection. We proposed that the protein 8b might enhance virulence in mice by affecting innate immune pathways. RNA-seq analysis showed that the innate immune signaling pathway was suppressed in the brains of mice infected with rJ2.2.8b. *In vitro* experiment was also confirmed the role of protein 8b on the innate immune response. However, subsequent experiments showed that rJ2.2 virus with aa 1–23 deletion had reduced mortality rate while did not affect IFN-I production in mouse brains. indicating although N-terminal domain is responsible for increased viral virulence, protein 8b potentially inhibits IFN-I response through its internal domains, which requires further investigation.

Our result showed that MRES-CoV protein 8b colocalized with ER and Golgi compartment. SARS-CoV accessory protein 6 which has the same cellular localization had been reported to be an amphipathic protein and its hydrophobic N-terminal region is sufficient to enhance virus replication (35). In this study, we found the α- helix C-region (aa 88–112) of protein 8b is critical for its subcellular location. However, the intracellular membrane location of protein 8b was not associated with virulence enhancement since deletion of C-terminus domain did not affect mouse survival after rJ2.2 infection. Interestingly, the distribution of Golgi and ER seem diffused in rJ2.2.8b Δ 88–112 infected cells which need further study.

In SARS-CoV, accessory protein 6 suppresses IFN-I production by interacting with karyopherin α2 via its cytoplasmic C-terminal region and inhibits STAT1 nuclear translocation (36). However, MERS-CoV accessory protein 8b did not change STAT1 translocation in 17CL-1 cells and BMMs (data not shown), suggesting different pathways used for IFN-I suppression between MERS-CoV protein 8b and SARS-CoV protein 6.

Unexpectedly, three truncated protein 8b proteins were detected in rJ2.2.8b infected cells, which could potentially reflect that the initiation of protein 8b protein translation could occur at different ATG codons on the 5′ end of the ORF8b mRNA. protein 8b might mainly initiated at the first two ATGs since more abundant protein bands were detected in rJ2.28b and MERS-CoV infected cells. The full-length protein 8b and truncated protein 8b with aa 1–8 deletion were responsible for the enhanced virulence *in vivo*, while protein 8b with 1–23 AA deletion expressed at minimal level did not cause lethal disease in MHV infected mice.

In summary, protein 8b was demonstrated to increase viral replication *in vivo* and *in vitro* and enhance virulence *in vivo*. protein 8b also inhibited IFN-I production, but the IFN-I antagonism effect did not contribute to its virulence enhancement *in vivo*. Similar to SARS-CoV protein 6 (37), MERS-CoV protein 8b had a modest effect on virus replication, while its expression in virus-infected cells resulted in greatly enhanced mortality in mice, suggesting a potentially important role when expressed in authentic MERS-CoV infected hosts.

## MATERIALS AND METHODS

### Cells, mice, and viruses.

17CL-1 (38) and HeLa-MHVR cells (39) (HeLa cells stably expressing MHV receptor carcinoembryonic antigen cell adhesion molecule isoform 1a [CEACAM1a]) were maintained in Dulbecco’s modified Eagle medium (DMEM) containing 10% fetal bovine serum (FBS), 1% P/S (penicillin-streptomycin), and 1% nonessential amino acids. L929 cells (40) were maintained in RPMI 1640 containing 10% FBS and 1% P/S, the cell-conditioned medium as a source for macrophage colony-stimulating factor [M-CSF]. Primary bone marrow cells were isolated from the hind legs of WT C57BL/6 mice as previously described (31), Bone marrow-derived macrophages (BMM) were cultured in DMEM supplemented with 10% FBS, 15% L929 cell-conditioned medium (40, 41), and 1% P/S. The cells were cultured 7 days and purity was determined by flow cytometry.

Specific pathogen–free 6–8-wk-old C57BL/6 mice were purchased from Hunan SJA Laboratory Animal Co. Mice were assessed for clinical disease using a previously described scale (5) as follows: 0, asymptomatic; 1, limp tailor slightly hunched; 2, wobbly gait or hunched or mild encephalitis; 3, hindlimb paresis or moderate encephalitis; 4, quadriparesis/paralysis or severe encephalitis; 5, moribund. In all experiments, mice were euthanized 21 days p.i. All animal studies were approved by the Institutional Animal Care and Use Committees of Guangzhou Medical University.

Recombinant viruses were constructed from rJ2.2, a virus derived from JHM strain as previously described (29). Briefly, MERS-CoV accessory proteins nucleotide sequence (with Flag tag in N-end and HA tag in C-end) was synthesized according to the reference sequence (GenBank accession number: NC_019843.3). The accessory protein nucleotide sequence was inserted into ORF4 of a plasmid containing genes rJ2.2. The recombinant plasmid was linearized and transcribed using T7 polymerase, then electrotransferred into feline cells (FCWF) previously infected with rJ2.2 and cocultured with DBT cells. Recombinant viruses were generated and purified by plaque assay. The presence of the introduced sequence was confirmed by sequence analysis.

### RT-PCR analysis.

RNA was harvest from 17Cl-1 or BMM cells or mice brains using TRIzol (Invitrogen), cDNA synthesis, and RT-PCR was performed as described (42). RNA transcriptional levels were normalized for the housekeeping gene (HPRT). All results are shown as a ratio to HPRT calculated as 2^-(CT (gene)-CT (HPRT/GAPDH))^. Primer sequences used in this study are as follow: MHV-N-Fwd: GACACAACCGACGTTCCTTT, MHV-N-Rev: TAGCAGGTGCAGACCTTCCT; TNF-Fwd: GAACTGGCAGAAGAGGCACT, Mouse-TNF-Rev: AGGGTCTGGGCCATAGAACT; Mouse-IFN-α-Fwd: TCCATCAGCAGCTCAATGAC, Mouse-IFN-α-Rev: AGGAAGAGAGGGCTCTCCAG; Mouse-IFN-β-Fwd: TCAGAATGAGTGGTGGTTGC, Mouse-IFN-β-Rev: GACCTTTCAAATGCAGTAGATTCA; Mouse-ISG15-Fwd: GGCCACAGCAACATCTATGA, Mouse-ISG15-Rev, CGCAAATGCTTGATCACTGT; Mouse-IL-6-Fwd: GAGGATACCACTCCCAACAGACC, Mouse-IL-6-Rev: AAGTGCATCATCGTTGTTCATACA; Mouse-IL1β-Fwd: ACTGTTTCTAATGCCTTCCC, Mouse-IL1β-Rev: ATGGTTTCTTGTGACCCTGA; Mouse-HPRT-Fwd: GCGTCGTGATTAGCGATGATG, Mouse-HPRT-Rev: CTCGAGCAAGTCTTTCAGTCC.

### Immunofluorescence assay (IFA).

The following antibodies were used: mouse anti-HA antibody (1:500, BioLegend), rabbit anti-Giantin antibody (1: 1000, Biolegend), rabbit anti-PDI antibody (1:50, CST), goat anti-Olig2 antibody (1:200, R&D), rabbit anti-GFAP antibody (1:200, Proteintech), rabbit anti-IBA1 antibody (1:250, Invitrogen), mouse anti-Neu-N antibody (1:100, Millipore), rabbit anti-HCoV-OC43-N antibody, Cy3-conjugated donkey anti-rabbit antibody (1:500, Jackson), Alexa Fluor 488-conjugated donkey anti-mouse antibody (1:500, Jackson), Alexa Fluor 555-conjugated donkey anti-mouse antibody (1:500, Jackson), Alexa Fluor 488 anti-HA.11 Epitope Tag Antibody (1:500, Biolegend), Cy3-conjugated donkey anti-goat antibody (1:500, Jackson).

17CL-1 Cells infected with the virus (MOI = 0.1) were fixed using 4% PFA in PBS, then the cells permeabilized with 0.2% Triton X-100 in PBS. Cells were incubated with primary antibody at RT for 1 h, and then incubated with second antibody at RT for 1 h, the nucleus was stained with DAPI. After three times wash with PBS, the coverslips were mounted on glass slides. Cells were examined by confocal microscopy (ZEISS).

### Western blot analysis.

The following antibodies were used: mouse anti-HA antibody (1:500, Biolegend), mouse anti-Flag antibody (1:1000, Sigma-Aldrich), mouse anti-MHV-N antibody (1:2000, prepared by our lab), Peroxidase AffiniPure Donkey Anti-Mouse IgG (H+L) (1:10000, Jackson). Rabbit anti-protein 8b antibody (1:1000, obtained by immunized with synthetic peptides TSTTLELDPKQHSHS [aa 33–47 of protein 8b]). 17CL-1 cells infected with recombinant rJ2.2 (MOI = 0.1), and total proteins were harvested at indicated time point. The proteins were resolved using reducing sodiumdodecyl sulfate-polyacrylamide gel electrophoresis (SDS-PAGE), and transferred to PVDC membranes. Membranes were washed once with PBST and incubated in blocking buffer (5% skim milk in PBST) at RT for 1.5 h. Then primary antibodies were incubated overnight at 4°C, after three times wash with TBST, the membranes were incubated with second antibody at RT for 1.5 h. The membranes were washed four times with TBST and were detected using an ECL plus detection kit.

### RNA-seq.

Total RNA was extracted from the brain of mice infected with recombinant virus at 3 days. p.i. and mRNA was enriched by magnetic beads with Oligo (dT). RNA quality was determined by NanoPhotometer (IMPLEN, CA, USA) and fragment size was analyzed using the 2100 RNA Nano 6000 assay kit (Agilent Technologies, CA, USA). The RNAseq libraries were prepared using NEBNext Ultra RNA Library Prep kit for Illumina (E7530L, NEB). Libraries were sequenced on an Illumina NovaSeq 6000 machine to obtain 150 bp paired-end reads. RNAseq reads were mapped to the mouse transcriptome (mm10) with Kallisto (version 1.15.0). Transcript abundances were normalized with Transcripts Per Million (TPM) mapped reads. Differentially expressed genes were identified by DESeq2 (v1.18.1) with fold change >2 and Benjamini–Hochberg false discovery rate adjusted *P* < 0.05.

### Statistical analysis.

A Student's *t* test was used to analyze differences in mean values between groups. All results are expressed as means ± standard errors of the means (SEM). *P* values of *≤*0.05 were considered statistically significant (*, *P* values of 0.05).

### Data availability.

Next-gen RNA sequence data supporting the findings in this study are available in in the Gene Expression Omnibus (GEO) database under accession number GSE188514.
